# Polygenic risk scores for severe psychiatric disorders in bipolar disorders: associations with the clinical and dimensional expression, interactions with childhood maltreatment and mediation models

**DOI:** 10.1038/s41398-025-03466-5

**Published:** 2025-07-25

**Authors:** Bruno Etain, Mohamed Lajnef, Ophélia Godin, Cynthia Marie-Claire, Frank Bellivier, Elisa Courtois, Violaine Latapie, Sébastien Gard, Raoul Belzeaux, Philippe Courtet, Caroline Dubertret, Emmanuel Haffen, Antoine Lefrere, Emilie Olie, Mircea Polosan, Paul Roux, Ludovic Samalin, Raymund Schwan, Marion Leboyer, Stéphane Jamain, on behalf of FondaMental Advanced Centers of Expertise in Bipolar Disorders (FACE-BD) Collaborators, PM Llorca, V. Barteau, S. Bensalem, H. Laouamri, K. Souryis, S. Hotier, A. Pelletier, L. Wuillaume, E. Bourdin, V. Hennion, E. Marlinge, J. Meheust, A. Richard, M. Carminati, B. Aouizerate, A. Desage, A. Jutant, K. Mbailara, I. Minois, L. Zanouy, AM Auxilia, M. Benramdane, L. Boukhobza, B. Deffinis, S. Denat, D. Ducasse, M. Gachet, F. Molière, L. Nass, G. Tarquini, M. Cermolacce, E. Moreau, H. Polomeni, F. Groppi, J. Baurberg, L. Lescalier, I. Murracioli, A. Surray, R. Cohen, G. Gross, T. Schwitzer, O. Wajsbrot-Elgrabli, T. Bougerol, B. Fredembach, Q. Denoual, A. Bertrand, A. Pouchon, G. Bonny, L. Brehon, L. Durand, V. Feuga, AM Galliot, N. Kayser, C. Passerieux, A. Aubin, I. Cussac, MA Dupont, J. Loftus, I. Medecin, N. Mazer, C. Portalier, P. Laurent, C. Beal, O. Blanc, T. Bonnet, D. Lacelle, M. Mennetrier, LM Vayssié

**Affiliations:** 1 Université Paris Cité, INSERM UMR-S 1144, Optimisation Thérapeutique en Neuropsychopharmacologie OTeN, Paris, France; 2 AP-HP, Groupe Hospitalo-Universitaire AP-HP Nord, DMU Neurosciences, Hôpital Fernand Widal, Département de Psychiatrie et de Médecine Addictologique, Paris, France; 3 Fondation FondaMental, Créteil, France; 4 Université Paris Est Créteil, INSERM U955, IMRB, Translational NeuroPsychiatry Laboratory, Créteil, France; 5 Centre Hospitalier Charles Perrens, Pôle de Psychiatrie Générale et Universitaire, Bordeaux, France; 6 Pôle Universitaire de Psychiatrie, CHU de Montpellier, IGF, Univ. Montpellier, CNRS, INSERM, Montpellier, France; 7 Department of Emergency Psychiatry and Acute Care, CHU Montpellier, IGF, Université Montpellier, CNRS, INSERM, Montpellier, France; 8 AP-HP, Groupe Hospitalo-Universitaire AP-HP Nord, DMU ESPRIT, Service de Psychiatrie et Addictologie, Hôpital Louis Mourier, Colombes, France; 9 Université Paris Cité, Inserm UMR1266, Sorbonne Paris Cité, Faculté de Médecine, Paris, France; 10 Université de Franche-Comté, UR LINC, Service de Psychiatrie de l’Adulte, CIC-1431 INSERM, CHU de Besançon, Besançon, France; 11 Pôle de Psychiatrie, Assistance Publique Hôpitaux de Marseille, Marseille, France; 12 INT-UMR7289, CNRS Aix-Marseille Université, Marseille, France; 13 Université Grenoble Alpes, Inserm, U1216, CHU Grenoble Alpes, Grenoble Institut Neurosciences, Grenoble, France; 14 Centre Hospitalier de Versailles, Service Universitaire de Psychiatrie d’Adultes et d’Addictologie, Le Chesnay; Université Paris-Saclay; Université de Versailles Saint-Quentin-En-Yvelines; DisAP-DevPsy-CESP, INSERM UMR1018, Villejuif, France; 15 Centre Hospitalier et Universitaire, Département de Psychiatrie, Université Clermont Auvergne, CNRS, Clermont Auvergne INP, Institut Pascal (UMR 6602), Clermont-Ferrand, France; 16 Université de Lorraine, Centre Psychothérapique de Nancy, Inserm U1254, Nancy, France; 17 AP-HP, Hôpitaux Universitaires Henri Mondor, Département Médico-Universitaire de Psychiatrie et d’Addictologie (DMUIMPACT), Fédération Hospitalo-Universitaire de Médecine de Précision en Psychiatrie (FHU ADAPT), Créteil, France; 18 Pôle de Psychiatrie, Centre Hospitalier Princesse Grace, Monaco, Monaco

**Keywords:** Bipolar disorder, Clinical genetics

## Abstract

Polygenic risk scores (PRSs) for several psychiatric disorders have been associated with the clinical presentation of bipolar disorder (BD). PRSs have also been suggested to moderate the associations between childhood maltreatment and BD severity. In this study, we investigated how PRSs for BD, schizophrenia, major depressive disorders (MDD) and attention-deficit/hyperactivity disorder (ADHD) might disentangle the clinical and dimensional heterogeneity of BD in a sample of 852 affected individuals. We used logistic and linear regressions, moderation and mediation models to test the associations between PRSs, dimensions in childhood/adulthood and clinical indicators of severity of BD. All models were adjusted for age, sex, BD type and depressive symptoms. None of the PRSs were significantly associated with the clinical expression of BD when considered in terms of mode of onset, course, or psychiatric comorbidities. Nevertheless, the PRS-ADHD significantly and positively correlated with the levels of childhood maltreatment, childhood ADHD symptoms, and of some adulthood measures (affective lability, impulsivity and hostility) with *p* values ranging from 3.10^−8^–4.10^−4^. None of the PRSs moderated the effects of childhood maltreatment on the clinical or dimensional variables. Mediation model suggested paths from both PRS-ADHD and PRS-MDD to childhood ADHD symptoms and childhood maltreatment. The links between PRS-ADHD to all adulthood dimensions were mediated by childhood ADHD symptoms (*p* < 0.002). In turn, some adulthood dimensions (mainly affect intensity and affective lability) were associated with the clinical severity of BD, as defined by rapid cycling, suicide attempts and anxiety disorders. In conclusion, this study disentangles the associations between the genetic liability for four psychiatric disorders and the clinical/dimensional heterogeneity of BD. We suggest a continuum from the genetic risk for ADHD and MDD through dimensions in childhood/adulthood to a severe/complex clinical expression of BD.

## Introduction

Genome-wide association studies (GWAS) in large samples of individuals suffering from psychiatric disorders have led to the development and use in clinical research of polygenic risk scores (PRSs). A PRS is an estimate of an individual’s genetic liability to a disease or a trait, calculated according to the genotype profile and based on summary statistics of GWAS data [1]. The classical PRS calculation combines the effect sizes of multiple single nucleotide polymorphisms (SNPs) from GWAS studies into a single aggregated score. A PRS is expected to reflect individuals’ disease risk [2].

In bipolar disorders (BD), beyond reflecting the individual risk, PRSs have also been suggested to be associated with the clinical heterogeneity of the disorder [2]. As examples, individuals with BD type 1 had, in average, a lower PRS for major depressive disorder (PRS-MDD) when compared to those with BD type 2 [3]. Psychotic symptoms in BD have been associated with a higher PRS for schizophrenia (PRS-SZ) [4–7], but also with a higher PRS for BD (PRS-BD) [8]. Suicidal ideation has been associated with a higher PRS-BD [3], and suicide attempts with a higher PRS-MDD [5]. Rapid cycling has been associated with a higher PRS for attention deficit with hyperactivity disorder (PRS-ADHD), with a higher PRS-MDD, but a lower PRS-BD [5, 9], or with a higher PRS-SZ [6]. Findings regarding age at onset of BD are conflicting, with an association with both PRS-MDD and PRS-SZ in some studies, while no association with PRS-BD or PRS-SZ was reported in other studies [6, 10, 11]. Altogether, these studies suggest that PRSs for several psychiatric disorders might be associated with the clinical presentation of BD. A recent review of 142 articles (of which 37 reported 167 analyses with multiple PRSs) concluded towards moderate to strong evidence for positive associations between the genetic risk of BD and the age at onset, the BD type, and the presence of psychotic symptoms [12].

PRSs have also been investigated as part of the postulated gene-environment interactions in BD. According to this model, a given PRS is supposed to moderate the association between a given environmental risk factor and the clinical presentation of BD. Although few studies have been published in the litterature, the three most striking examples combined the study of PRSs and childhood maltreatment in BD. The first one showed an interaction between PRS-BD and childhood maltreatment on the risk of rapid cycling, with no further interactions observed for other clinical characteristics (age at onset, suicide attempts, lifetime number of mood episodes, mixed or psychotic symptoms, substance use disorders) [13]. The second observed that both groups with more adverse childhood experiences and a higher PRS-BD were interactively associated with an earlier age at onset of BD [14]. The most recent study failed to identify any interaction between adverse childhood experiences and any of the calculated PRSs (BD, SZ, MDD and ADHD) on age at onset, psychotic symptoms, suicidal ideation, or rapid cycling [9]. Further studies are therefore required to explore any potential interactions between PRSs and childhood maltreatment on the clinical expression of BD.

PRSs might also be relevant when used with dimensional measures of psychopathology that are core in BD. This has never been investigated in BD, since previous studies focused on the clinical expression of BD mostly in terms of course and/or associated conditions. Dimensions of psychopathology include - for instance - affective lability, affect intensity, impulsivity and hostility that all demonstrated associations with a more severe/complex clinical expression of BD [15–17]. Interestingly, these dimensions are not specific to BD, but rather transdiagnostic, since shared with ADHD, psychosis or major depression for affective lability [18–20] or for impulsivity [21, 22] and therefore are relevant to be investigated with PRSs for several psychiatric disorders.

More complex models would therefore benefit from the investigation of potential missing links, i.e., how PRSs might take place into broader mediation models going from PRSs through childhood dimensions (maltreatment or early symptoms), to dimensions of psychopathology in adulthood and finally to the clinical expression of BD. A higher PRS-ADHD has been associated with a higher number of adverse childhood experiences, such an association with childhood adversity not being observed with PRS-BD, PRS-SZ nor PRS-MDD [9]. Associations between both PRS-ADHD and PRS-MDD and a higher risk of experiencing different type of childhood abuse (physical/emotional abuse, physical assault, and sexual abuse) have also been reported [23]. In turn, adulthood dimensions might mediate the effects of childhood maltreatment on the clinical expression of BD, especially suicide attempts, substance misuse, age at onset, or rapid cycling [24–26]. Therefore, we may postulate a continuum between the genetic risk for psychiatric disorders to the dimensional/clinical expression of BD, through childhood dimensions of maltreatment for instance.

The aim of this study was therefore to disentangle the role of PRSs for four psychiatric disorders (BD, SZ, MDD and ADHD) on the clinical and dimensional expression of BD, using both associations, moderation and mediation models. Our first aim was to test whether PRSs were associated with the clinical expression of BD and/or with childhood maltreatment and dimensions of psychopathology in adults. The second aim was to replicate a moderation effect of PRSs on the association between childhood maltreatment and the clinical expression of BD, and to extend the investigation to the dimensional expression of BD. The third aim was to integrate findings into a broader mediation model to visualize the paths going from PRSs to adulthood dimensions and clinical variables and assess whether some mediation effects of childhood dimensions (childhood maltreatment and childhood ADHD symptoms) were observed.

## Material and methods

### Participants

The sample included individuals who were clinically assessed in the French network of FondaMental Advanced Centers of Expertise in Bipolar Disorders (FACE-BD). This network is supported by the French Ministry of Health and has been developed under the aegis of the non-profit Fondation FondaMental, to offer specialized and personalized care for individuals with BD. Nine hundred and ninety outpatients aged 16 years or older, diagnosed with BD according to DSM-IV criteria (type 1, type 2 and not otherwise specified: NOS) [27] underwent a standardized clinical assessment. Eleven expert centers in France (Bordeaux, Créteil, Montpellier, Grenoble, Nancy, Marseille, Paris, Versailles, Clermont-Ferrand, Colombes and Besançon) used the same comprehensive clinical assessments, described in detail elsewhere [28]. The assessment protocol was approved by the institutional review boards (Comité de Protection des Personnes Ile de France V and VI). Written informed consent was obtained from all participants as part of the PsyCohBP (reference ID RCD: 2013-A01375-40) et Biobanque (reference ID RCB : 2013-A01286-39) research protocols.

### Clinical assessment

At inclusion, a multidisciplinary team (nurses, psychiatrists and psychologists) interviewed participants using the SCID [29] to confirm the diagnosis of BD and systematically collected information related to socio-demographic characteristics, characteristics at onset (age at, polarity of and psychotic symptoms during the first mood episode), course of BD (lifetime number of mood episodes, rapid cycling) and psychiatric associated conditions (lifetime suicidal attempt, anxiety disorders, alcohol use disorder (AUD) and cannabis use disorder (CUD)). Baseline depressive and (hypo)manic symptoms were assessed using the Montgomery Asberg Depression Rating Scale (MADRS) [30] and the Young Mania Rating Scale (YMRS), respectively [31]. Current psychotropic treatments at inclusion (lithium, anticonvulsants, atypical antipsychotics, antidepressants) were recorded.

### Dimensional assessment

Several dimensions were assessed using self-administered instruments. Two questionnaires were used to assess events and symptoms during childhood. We used the Childhood Trauma Questionnaire (CTQ) to measure childhood maltreatment [32] and the Wender Utah Rating Scale (WURS) that has been originally designed as an ADHD screening tool, but whose factorial structure captures broader childhood dimensions (impulsivity/temper, inattentiveness and mood lability) experienced by individuals before the age of 12 [33]. We used four self-reports to assess adulthood dimensions of affect intensity, affective lability, impulsivity and hostility. The Affect Intensity Measure (AIM) quantifies affect intensity as responses to a given level of emotion-provoking stimulation [34]. The Affective Lability Scale (ALS) [35] measures the proneness to rapidly switching from one emotion to another during a short period of time. The Barratt Impulsiveness Scale (BIS) [36] measures impulsiveness as a stable characteristic. The Buss-Durkee Hostility Inventory (BDHI) measures individual differences in traits of general aggression and hostility [37], with two scores of hostility as described by the validation article: attitudinal hostility (resentment, suspicion and guilt) and motor hostility (assault, indirect hostility, verbal hostility and irritability). To reduce the number of variables to be investigated, we restricted the analyses to total scores for all these dimensions, except for the BDHI for which we used the two scores.

### DNA extraction from blood samples and genotyping

Genomic DNA was isolated from venous blood sample. DNA extraction was performed as previously described [38] and using prepIT® L2P kit (DNA genotek, Kanata, ON, Canada), following manufacturer’s instructions. Genotyping of ~660,000 SNPs was performed using Infinium Global Screening Array (GSA)-24 v2.0 or v3.0 (Illumina Inc., San Diego, CA, U.S.A.). Quality control of genotypic data was performed using the PLINK v.1.9 [39] as previously described [38] and following recommendation for PRS calculation [1]. Briefly, samples with sex discordance between genetic and clinical recording, with an abnormal heterozygosity rate (heterozygosity rate greater or lower than two standard deviations of the mean), or with a low genotyping rate (<0.98) were excluded of analyses. Ancestry of participants has been approximated using genotypic data of the 1000 Genomes Project populations [40] and only participants with a European ancestry (*N* = 852) have been included in the analyses. SNPs with a genotyping rate of less than 0.99, a Hardy-Weinberg equilibrium p-value of less than 10^−6^, and a minor allele frequency of less than 0.01 were removed. Remaining SNPs were subsequently used for imputation using the Michigan Imputation Server [41]. Phasing and imputation were conducted using Eagle v2.4 and minimac4, respectively, using the Haplotype Reference Consortium (HRC) r1.1 2016 reference panel [42]. After imputation, only biallelic SNPs with a MAF greater than 0.01 in the reference panel and an imputation score greater than 0.9 were used for PRSs calculation.

### Polygenic risk scores calculations

A Bayesian method was used to calculate PRS-BD, PRS-SZ, PRS-MDD and PRS-ADHD. They were calculated for each individual using the LDpred2 software with the LDpred2-auto option and the HapMap3+ set of variants provided by the authors [43], and were based on PGC summary statistics for BD [44], SZ [45], MDD [46], or ADHD [47] with European individuals only. This software includes SNPs according to their linkage disequilibrium and estimates their effect using a Bayesian framework, resulting in a better predictive value than the PRS calculation based on selected p-value thresholds, which risks overfitting genetic effects. For the analyses, PRSs have then been standardized to have a mean of 0 and a standard deviation of 1.

### Statistical analyses

Data were mainly analyzed using SPSS. Categorical variables were reported as number and percentages. Continuous variables were reported as means (standard deviations) or medians (interquartile range: IQR). Normality of distribution was checked of using Skewness and Kurtosis values. We used Pearson or Spearman correlation tests to investigate associations between two continuous variables and t-tests, Mann-Whitney tests or Kruskal-Wallis tests to investigate associations between categorical and continuous variables.

We used logistic (for binary variables), linear (for age at onset) or negative binomial (for lifetime number of mood episodes) regression models with a given clinical or dimensional variable as the dependent variable and the four PRSs as independent variables, with an adjustment for age, sex, BD type (type 1 *versus* type 2 + type NOS) and MADRS score. Analyses were adjusted for the first six principal components of the population’s genetic substructure). Collinearity was checked using tolerance and Variance Inflation Factor (VIF).

To test the potential interactions between CTQ, PRSs (moderators) and the clinical/dimensional variables, we used the Process macro v4.1 for SPSS [48]. Process allows for both dichotomous and continuous outcomes and estimates the coefficients of the model accordingly using regression. As Process includes bootstrapping, it is a well-suited method for analyzing variables that are not normally distributed.

We finally used Mplus 8.8 [49] to test for a mediation effect of childhood dimensions (CTQ and WURS) on the links between PRSs and adulthood dimensions and clinical variables. Path analyses from PRSs through childhood dimensions (CTQ and WURS) to the dimensional measures (AIM, ALS, BIS, BDHI) and the clinical expression of BD were performed using MLR estimator (maximum likelihood estimation with robust standard errors) which provides maximum likelihood parameter estimates with standard errors and a chi-square test statistic (when applicable) that are robust to non-normality and non-independence of observations. The model examined both direct and indirect associations between PRSs and the variables in adulthood. The model also estimated correlations between (i) predictors, (ii) mediators and (ii) the dimensional/clinical outcomes. A path diagram representation of the model was drawn with straight single-headed arrows represented regression pathes and two-headed arrows represented associations/correlations. An iterative procedure allowed the selection of the best-fitting model. The path analysis started with a saturated model in which all variables were interrelated. Then non-significant path correlations/associations were gradually excluded until a good-fitting model was reached by utilizing modification indices (MI) to enhance the model’s fit. Goodness of fit was reported using standard indices: Comparative Fit Index (CFI), Tucker and Lewis Index (TLI), Root Mean Square Error of Approximation (RMSEA) and standardized root-square residual (SRMR). Rules of thumb for determining acceptable model fit were CFI or TLI values of 0.90 or above, RMSEA values close to 0.05 or below and a SRMR values of 0.08 or less. All path coefficients and correlations are reported as standardized estimates with corresponding *p* values.

We applied a Bonferroni’s correction for multiple testing and findings obtained with a nominal *p* < 0.0006 were considered as significant (given 84 tests being performed). Since the final mediation model was exploratory, no correction for multiple testing was applied for this model.

## Results

### Sample description

We included 852 participants with a European ancestry in our analyses. Most participants were females (63%). The median age at inclusion was 33 years old (IQR: 26–44). Individuals were mostly diagnosed with BD type 1 (48%) or BD type 2 (46%). Most participants had low depressive and manic symptoms at inclusion (respectively median MADRS score = 8 (IQR: 3–15), and median YMRS score = 1 (IQR: 0–3)). Age at onset had a median of 21 (IQR: 17–26). Most participants had a depressive polarity at onset (72%), and a non-psychotic mode at onset (85%). The median lifetime number of mood episodes was 6 (IQR: 3–10), with rapid cycling for 17% of the participants. Comorbidities were frequent: lifetime anxiety disorders (49%), current smoking (46%), lifetime suicide attempt (37%), lifetime alcohol use disorder (27%) and lifetime cannabis use disorder (23%). Further information about the characteristics of the sample, medications at inclusion, scores for the different dimensional scales and values for the four PRSs are given in Table 1.

**Table 1:** Sample description (852 individuals with bipolar disorders).

Variables	N	%	Median	IQR	Missing
Socio-demographic and clinical characteristics					
Sex (females)	538	63.1%			0
Age at inclusion			33	26–44	0
BD type 1	409	48.0%			0
BD type 2	391	45.9%			
BD type NOS	52	6.1%			
MADRS			8	3–15	3
YMRS			1	0–3	6
Age at BD onset			21	17–26	37
Polarity at onset (depressive)	592	71.8%			27
Psychotic symptoms at onset	128	15.5%			27
Duration of illness			11	5–18	38
Number of lifetime mood episodes			6	3–10	34
Number of hospitalisations	793		2	1–3	59
Lifetime rapid cycling	128	16.8%			90
Lifetime suicidal attempt	309	37.2%			22
Comorbidities					
Current smoker	378	46.4%			38
Lifetime alcohol use disorder	201	26.6%			97
Lifetime cannabis use disorder	173	22.9%			97
Lifetime anxiety disorders	362	48.8%			111
Medications at inclusion					
Lithium	277	39.9%			158
Anticonvulsants	358	51.5%			157
Atypical antipsychotics	286	43.0%			187
Antidepressants	236	35.5%			187
Dimensionnal assessments					
CTQ total score			37	31–47	52
Childhood ADHD symptoms (WURS)			29	17–46	61
Affect intensity measure (AIM)			3.78	3.33–4.22	62
Affective lability scale (ALS)			1.30	0.80–1.70	72
Barrat impulsivity scale (BIS)			66	59–75	63
BDHI Attitudinal component			8	4–11	90
BDHI Motor component			20	15–27	96
Polygenic Risk Scores (PRS)					
PRS-BD			0.019	−0.660–0.623	0
PRS-SZ			0.013	−0.693–0.715	0
PRS-MDD			0.040	−0.675–0.692	0
PRS-ADHD			0.006	−0.692–0.665	0

*IQR* interquartile range, *BD* bipolar disorder, *NOS* not otherwise specified, *MADRS* montgomery asberg depression rating scale, *YMRS* young mania rating scale, *BDHI* buss durkee hostility inventory, *BIS* barrat impulsivity scale, *CTQ* childhood trauma questionnaire, *WURS* wender utah rating scale, *PRS* polygenic risk score, *SZ* schizophrenia, *MDD* major depressive disorder, *ADHD* attention deficit with hyperactivity disorder.

Several significant positive correlations were observed between PRSs (see Supplementary Table S1). As previously reported [50], the two highest correlation coefficients were observed between PRS-MDD and PRS-ADHD (rho = 0.37, *p* = 9.10^−30^) and between PRS-BD and PRS-SZ (rho = 0.34, *p* = 1.10^−24^).

### Association between PRSs, clinical expression of BD and comorbidities

Multivariable regression models were used for each clinical variable, including the four PRSs and age, sex, BD type and MADRS as covariates (with an adjustment for the first six principal components of the population’s genetic substructure). We explored the following clinical characteristics: BD type, age at onset, polarity at onset, psychotic symptoms at onset, lifetime number of mood episodes, rapid cycling. We explored the following associated conditions and comorbidities: suicidal attempt, current smoking, lifetime alcohol use disorder and cannabis use disorder, lifetime anxiety disorders.

A higher PRS-MDD was associated with rapid cycling (*p* = 0.025), suicide attempt (*p* = 0.002) and lifetime alcohol use disorders (*p* = 0.019). PRS-BD was higher in BD type 1 (*p* = 0.033). No multicollinearity was detected. No association remained significant after correction for multiple testing. Results are presented in details in Supplementary Table S2a–k.

### Association between PRSs and dimensional assessments in BD

Of note, childhood and adulthood dimensions significantly and positively correlated together (*p* values ranging from 7.10^−80^–1.10^−8^) (see Supplementary Table S3).

**Fig. 1:**
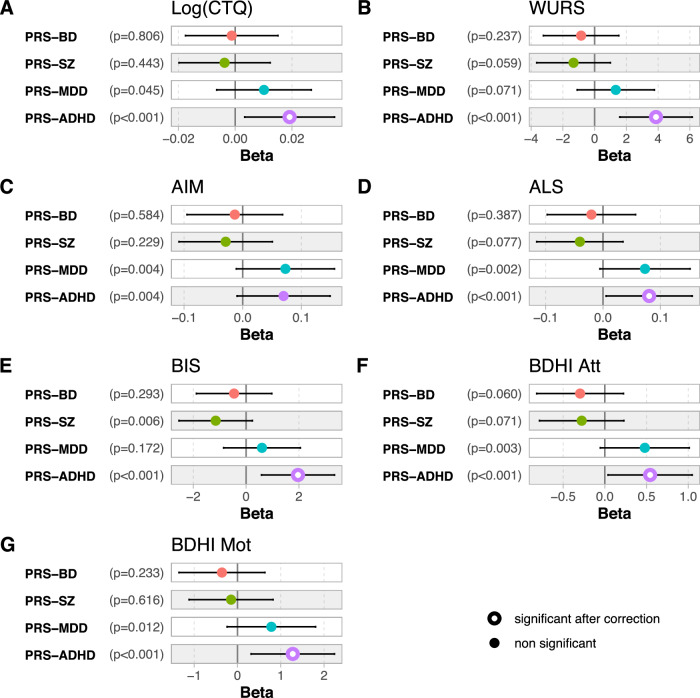
Associations of each dimensional assessment in association with the four PRSs in multivariable analyses adjusted for age, se, bipolar disorder type, depressive symptoms and the six first components of the population’s genetic substructure. Beta represent unstandardized coefficients obtained from the linear regressions of each dimension in association with the four PRS as dependent variables. adjusting for age, sex, BD type and MADRS score. All models were adjusted for the first six principal components of the population’s genetic substructure. For a question of clarity. unstandardized coefficients for age, sex, BD type, MADRS, and PCs are not displayed into the figures. Nominal p-values are indicated in brackets. **A** CTQ childhood trauma questionnaire (Log: log10 transformed), **B** WURS wender utah rating scale, **C** AIM affect intensity measure, **D** ALS affective lability scale, **E** BIS barrat impulsivity scale, **F** BDHIAtt buss durkee hostility inventory attitudinal component; **G** BDHIMot buss durkee hostility inventory motor component. PRS polygenic risk score, BD bipolar disorder, SZ schizophrenia, MDD major depressive disorder, ADHD attention deficit with hyperactivity disorder.

To explore whether PRSs were associated with the different dimensions, linear regression models were used, with the four PRSs as independent variables, and age, sex, BD type and MADRS score as covariates (with an adjustment for the first six principal components of the population’s genetic substructure). CTQ score was log transformed for the analyses. Results are detailled in Supplementary Table S4a–f. A higher PRS-ADHD was associated with higher levels of childhood maltreatment (*p* = 7.10^−5^), of childhood ADHD symptoms (*p* = 3.10^−8^), and higher levels of affective lability (*p* = 4.10^−4^), impulsivity (*p* = 3.10^−6^), attitudinal hostility (*p* = 4.10^−4^), and motor hostility (*p* = 1.10^−5^). These results are summarized in Fig. 1. No multicollinearity was detected based on variance inflation factor. Some associations that did not remain significant after correction for multiple testing were observed between PRS-MDD and childhood maltreatment (*p* = 0.045), affect intensity (*p* = 0.004), affective lability (*p* = 0.002), attitudinal hostility (*p* = 0.003) and motor hostility (*p* = 0.012), between PRS-ADHD and affect intensity (*p* = 0.004), and between PRS-SZ and impulsivity (*p* = 0.006).

### Interactions between PRSs and childhood maltreatment on the expression of BD

Given previous evidence that childhood maltreatment was associated with a more severe expression of BD, we first tested associations between the CTQ score, clinical variables and comorbidities (see Supplementary Table S5a, b for details). Childhood maltreatment was associated with a lower age at onset (*p* = 0.006), to psychotic symptoms at onset (*p* = 0.03), rapid cycling (*p* = 0.002), alcohol use disorder (*p* = 0.002) which were not significant after correction for multiple testing. Two associations remained significant after correction for multiple testing, the association between childhood maltreatment and suicide attempt (*p* = 8.10^−9^) and anxiety disorders (5.10^−6^). We therefore used CTQ as a predictor and each PRS as a moderator for these two latter clinical outcomes in four separate models of moderation (one models per outcome, each one using one PRS). We found no interaction (see Table S6).

Regarding dimensional assessments, since the CTQ score was correlated with all other dimensions (see Supplementary Table S3), we used CTQ as a predictor and each PRS as a moderator for six dimensional outcomes in separate models of moderation. None of the PRSs moderated the links between CTQ and other dimensions (see Table S6).

### Mediation model

**Fig. 2:**
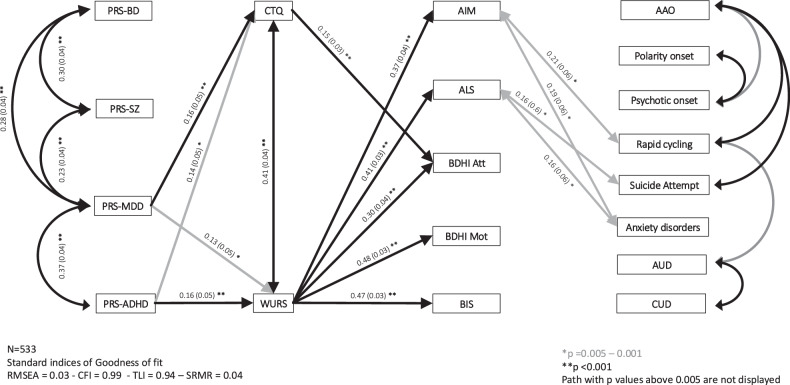
Path analysis diagram including PRS-BD. PRS-ADHD. childhood measures. adulthood dimensions and clinical variables. adjusted for age. sex. BD type and MADRS score (*n* = 533). Single-headed arrows represent regression paths and double-headed (straight and curved) arrows represent correlations. All path coefficients and correlations are reported as standardized estimates. The level of significance of path coefficients and correlations is given both by the type of arrow and the number of stars. For reasons of clarity. the paths corresponding to *p* values > 0.05 were not included in the path diagram. For reasons of clarity. the paths between adulthood dimensions are not displayed (all *p* < 0.001). For reasons of clarity. only significant associations in-between clinical variables are displayed (*p* < 0.005). PRS polygenic risk score, BD bipolar disorder, ADHD attention deficit with hyperactivity disorder, CTQ childhood trauma questionnaire, WURS wender utah rating scale (childhood ADHD symptoms), AIM affect intensity measure, ALS affective lability scale, BDHI Att buss durkee hostility inventory attitudinal component, BDHI Mot buss durkee hostility inventory motor component, BIS barrat impulsivity scale, RMSEA root mean square error of approximation, CFI comparative fit index, TLI tucker and lewis index.

Finally, we constructed an exploratory mediation model to explore the links between the four PRSs, CTQ and WURS as mediators and both adulthood dimensions and clinical variables as outcomes. We adjusted the model for age, sex, BD type and MADRS scores. The number of mood episodes was not included in the model because of a highly skewed distribution. The mediation model included data for 533 individuals without any missing variables. The final model has no evidence of poor fit as indicated by a CFI = 0.99, a TLI = 0.94, a RMSEA = 0.03 and a SRMR = 0.04. Fig. 2 indicates standardized estimates of paths and correlations/associations selected based on *p* values < 0.005.

No significant path emerged from PRS-BD nor PRS-SZ. PRS-ADHD and PRS-MDD were both associated with higher scores at the CTQ and the WURS (*p* < 0.005). The WURS was significantly associated with all adulthood dimensions (all *p* < 0.001), while the CTQ was associated only with BDHI Attitudinal (*p* < 0.001). The CTQ mediated the links from PRS-MDD and PRS-ADHD to BDHI Attitudinal (respectively *p* = 0.007 and *p* = 0.009). The WURS mediated the links from PRS-ADHD to all dimensions (all *p* values < 0.002). To a lesser extent (all *p* values around 0.01), the WURS also mediated the links from PRS-MDD to all adult dimensions. A summary of all specific indirect effects from PRS-ADHD and PRS-MDD to CTQ and WURS to dimensions is presented in Table S7.

The model further identified associations between a higher ALS and suicide attempt (*p* = 0.001), and anxiety disorders (*p* = 0.003), and between a higher AIM and anxiety disorders (*p* = 0.001) and rapid cycling (*p* = 0.001). Some associations of smaller magnitude (*p* values ranging from 0.001 and 0.05) were observed between AIM and age at onset (*p* = 0.03), ALS and age at onset (*p* = 0.02), polarity at onset (*p* = 0.02), and rapid cycling (*p* = 0.01), between BDHI Attitudinal and anxiety disorders (*p* = 0.02), between BDHI Motor and psychotic onset (*p* = 0.006), and between BIS and cannabis use disorders (*p* = 0.009) (not diplayed on Fig. 2 for a question of clarity).

The model finally identified significant associations between several clinical variables with the strongest associations being observed between age at onset, rapid cycling and suicide attempt, between polarity and psychosis at onset, and between alcohol use disorders and cannabis use disorders (*p* < 0.005).

## Discussion

This study aimed at disentangling the associations between PRSs for four psychiatric disorders, the clinical expression of BD, and a range of affective dimensions. Our findings suggest a strong influence of PRS-ADHD over other PRSs and a continuum from the genetic risk for ADHD through measures in childhood and adulthood to a severe/complex clinical expression of BD.

We did not replicate previous associations between the PRSs and the clinical expression of BD, in terms of BD type, age at onset or psychosis. We found some associations between PRS-MDD, rapid cycling, suicide attempt and alcohol use disorders, and between PRS-BD and BD type 1, however not significant after correction for multiple testing. Several factors may explain this lack of replication. First, we cannot exclude a lack of power in a moderate sample size. Indeed, previous studies have included between 255 and 12,977 individuals, and five out of the nine published studies have a sample size above 1800 individuals [3–11]. Some studies used p-value threshold-based PRSs, while others (like ours) used Bayesian methods for calculating PRSs [3, 5, 6, 9]. Different calculations of PRSs may explain inconsistent findings. Our sample may also be of moderate severity given the low scores of depression and mania at inclusion and a low median number of hospitalizations. Finally, our sample includes a substantial proportion of BD type 2 (46%), which contrasts with previous studies that included a majority of (or exclusively) BD type 1.

We also did not replicate the initial reports about a moderation effect of some PRSs on the associations between childhood maltreatment and the clinical outcomes of BD. Here again, the sample sizes, the methods for PRSs calculation and the GWAS summary statistics used in earlier studies may explain differences between findings. Interestingly, our study design (*n* = 852 - four Bayesian PRSs) is similar to the one used in the latest study published so far (*n* = 885 - four Bayesian PRSs) showing no significant interaction between adverse childhood events and any of the PRSs in predicting selected BD sub-phenotypes (age at onset, psychotic symptoms, suicidal ideation, rapid cycling) [9]. Overall, this may suggest that PRSs for BD, schizophrenia, MDD or ADHD are unlikely to moderate the effect of early life adversity on the clinical severity of BD. However, this issue requires further investigation given the low number of published studies.

Given the dimensional assessments available in this sample, we propose a new way to disentangle the clinical expression of BD using a mediation model. The inclusion into the analyses of several childhood (maltreatment and ADHD symptoms) and adulthood dimensions (affect intensity, affective lability, impulsivity, hostility) that are strongly associated with BD offers some interesting preliminary findings. We suggest that both PRS-ADHD and PRS-MDD were associated with higher exposure to childhood maltreatment and higher levels of childhood ADHD symptoms, these latter leading to greater affect dysregulation and impulsivity/hostility in adulthood, that in turn (mainly for affective lability and affect intensity) were associated with several clinical indicators of BD severity (anxiety disorders, rapid cycling and suicide attempt). Two strengths of the analyses were the covariation by age, sex, BD type and depressive symptoms (that may confound some of the associations) and the comprehensive model proposed. Nevertheless, some limitations should be mentioned: the final sample size for the mediation model was around 500 individuals (due to missing data) and we did not apply any correction for multiple testing for this specific model since exploratory. Nevertheless, the findings obtained with this mediation model deserve some comments.

First, both PRS-MDD and PRS-ADHD was associated with the two childhood measures. This was expected for the association between PRS-ADHD and the WURS, that is designed to screen childhood ADHD symptoms. Our mediation model also identified links from both PRS-ADHD and PRS-MDD to the CTQ which is consistent with previous studies [9, 23]. Of note, one of the two previously published studies identified associations between the four PRSs (BD, SZ, MDD and ADHD) and physical/emotional abuse, while associations with sexual abuse were observed only for PRS-ADHD and PRS-MDD [23]. We cannot exclude a lack of power in our study since the mentioned previous study had a much larger sample (more than 10,000 participants). Nevertheless, this study included only white non-Hispanic women, while our sample consisted in both men and women who may differ both for PRS-ADHD and PRS-MDD, but also for the exposure to sexual abuse. Moreover, we used only the CTQ total score and did not further explore the different subscores for emotional, physical or sexual abuses. As a hypothesis, we may argue that the PRS-ADHD increased some externalized symptoms of motor impulsivity, resulting in a greater likelihood to be exposed to harsh discipline (emotional abuse and possibly physical abuse).

Second, strong associations were observed from the WURS to all dimensions in adulthood, that - in turn - were associated with some indicators of more severe/comorbid BD. These findings suggest a continuum between childhood ADHD symptoms and adulthood affect dysregulation and impulsivity/hostility. We previously demonstrated that childhood ADHD symptoms are strongly associated with BD when compared to healthy controls and that individuals with BD and a higher WURS score had an earlier age at onset, and increased risks for suicidal behaviors and polysubstance misuse [51]. In the same article, we also suggested that the WURS does not solely screen for ADHD specific symptoms, since a factor analysis identified three factors (‘impulsivity/temper’, ‘inattentiveness’, but also ‘labile mood/low self-esteem’). Since we did not include these three factors in the mediation model, but instead the WURS total score, we cannot infer that any specific factor contributes to this potential continuum between the WURS and the adulthood measures. The present mediation model also replicates some of the findings of the one we previously proposed in a smaller independent sample (*n* = 485) (but not including PRSs) showing that affective dysregulation was associated with high risk for lifetime presence of suicide attempts [24].

The major strengths of this study are the dimensional assessments that complete the clinical assessment, the use of four PRSs and the use of mediation models to better understand the links from the genetic risks to measures both in childhood and adulthood and the severity of the clinical expression of BD. Some limitations should be mentioned. The sample size was moderate which may hamper the likelihood to identify some associations with smaller effects sizes. All data obtained for this study were based on retrospective self-reports which does not exclude memory biases for childhood measures, but also misestimation for adulthood dimensions or desirability bias. The clinical interviews, although performed with a validated standardized instrument, may also not totally exclude recall bias (especially for mode of onset of BD). Due to missing variables, the final mediation model was performed in 533 individuals with complete data and this may result in a lack of power.

## Conclusion

This study disentangles the associations between PRSs for four psychiatric disorders (BD, SZ, MMD and ADHD) and the clinical/dimensional expression of BD. We suggest a strong influence of PRS-ADHD over other PRSs on both childhood dimensions (maltreatment and ADHD symptoms) and adulthood dimensions (affect intensity/lability and impulsivity/hostility), with further associations mostly between affect intensity/lability and some indicators of severity/comorbidity of BD (rapid cycling, suicide attempt and anxiety disorders). Taken together, these findings suggest a continuum from the genetic risk for ADHD through dimension of psychopathology in childhood/adulthood to a severe/complex clinical expression of BD. Replications are required in larger independent samples of individuals with BD.

## Supplementary information

Supplementary tables

## Data Availability

The data that support the findings of this study are available from the corresponding author upon reasonable request.
