# Supplementary material for: Polygenic risk scores for severe psychiatric disorders in bipolar disorders: associations with the clinical and dimensional expression, interactions with childhood maltreatment and mediation models
Source: Transl Psychiatry. 2025 Jul 25;15:256. doi: 10.1038/s41398-025-03466-5 (PMC12297605; doi:10.1038/s41398-025-03466-5)
Supplement: Supplementary file 1 — Supplementary tables [file 41398_2025_3466_MOESM1_ESM.docx]

**Supplementary table S1: Pearson correlations between PRSs (n=852)**

|  |  | PRS-SZ | PRS-MDD | PRS-ADHD |
| --- | --- | --- | --- | --- |
| PRS-BD | r | 0.34 | 0.25 | 0.08 |
|  | p | **1.10^-24^** | **1.10^-13^** | 0.015 |
| PRS-SZ | r |  | 0.21 | 0.08 |
|  | p |  | **9.10^-10^** | 0.025 |
| PRS-MDD | r |  |  | 0.37 |
|  | p |  |  | **9.10^-30^** |

*PRS: polygenic risk score. BD: bipolar disorder. SZ: schizophrenia. MDD: major depressive disorder. ADHD: attention deficit with hyperactivity disorder.*

*r: Pearson correlation coefficient*

***In bold: p values significant after correction for multiple testing (p<0.0006)***

**Supplementary tables S2a to S2k: Associations between PRSs, clinical characteristics and comorbidities of BD (multivariate analyses)**

**S2a) BD type (type 1 versus type 2+non specified) (n=849)**

| Variables | Beta | Standard Error | Wald | df | p |
| --- | --- | --- | --- | --- | --- |
| Age | -0.001 | 0.006 | 0.008 | 1 | 0.927 |
| Sex | 0.429 | 0.149 | 8.302 | 1 | 0.004 |
| MADRS | 0.043 | 0.008 | 25.953 | 1 | **3.10^-7^** |
| PRS-BD | -0.170 | 0.080 | 4.542 | 1 | 0.033 |
| PRS-SZ | -0.099 | 0.077 | 1.661 | 1 | 0.198 |
| PRS-MDD | 0.129 | 0.082 | 2.450 | 1 | 0.118 |
| PRS-ADHD | 0.025 | 0.077 | 0.107 | 1 | 0.743 |
| Constant | -1.367 | 1.950 | 0.492 | 1 | 0.483 |

**S2b) Age at onset of bipolar disorder (n=812)**

| Variables | Unstandardized  Beta | Standard  Error | Standardized  Beta | t | p |
| --- | --- | --- | --- | --- | --- |
| Age | 0.420 | 0.020 | 0.591 | 20.680 | 2.10^-76^ |
| Sex | -0.948 | 0.513 | -0.053 | -1.849 | 0.065 |
| BD type | 0.029 | 0.505 | 0.002 | 0.058 | 0.954 |
| MADRS | -0.058 | 0.028 | -0.061 | -2.092 | 0.037 |
| PRS-BD | -0.249 | 0.272 | -0.028 | -0.917 | 0.360 |
| PRS-SZ | -0.274 | 0.266 | -0.032 | -1.029 | 0.304 |
| PRS-MDD | -0.201 | 0.283 | -0.023 | -0.713 | 0.476 |
| PRS-ADHD | 0.279 | 0.267 | 0.032 | 1.042 | 0.298 |
| Constant | 15.547 | 6.813 |  | 2.282 | 0.023 |

**S2c) Polarity at onset (hypo-manic versus depressive) (n=823)**

| Variables | Beta | Standard Error | Wald | df | p |
| --- | --- | --- | --- | --- | --- |
| Age | -0.002 | 0.007 | 0.050 | 1 | 0.823 |
| Sex | -0.497 | 0.171 | 8.448 | 1 | 0.004 |
| BD type | -1.357 | 0.176 | 59.786 | 1 | **1.10^-14^** |
| MADRS | -0.035 | 0.011 | 11.137 | 1 | 0.0008 |
| PRS-BD | 0.058 | 0.094 | 0.373 | 1 | 0.542 |
| PRS-SZ | 0.042 | 0.091 | 0.211 | 1 | 0.646 |
| PRS-MDD | 0.046 | 0.096 | 0.230 | 1 | 0.631 |
| PRS-ADHD | 0.073 | 0.091 | 0.638 | 1 | 0.425 |
| Constant | 0.992 | 2.225 | 0.199 | 1 | 0.656 |

**S2d) Psychotic symptoms at onset (n=823)**

| Variables | Beta | Standard Error | Wald | df | p |
| --- | --- | --- | --- | --- | --- |
| Age | -0.001 | 0.009 | 0.027 | 1 | 0.870 |
| Sex | -0.155 | 0.212 | 0.534 | 1 | 0.465 |
| BD type | -1.966 | 0.266 | 54.716 | 1 | **1.10^-13^** |
| MADRS | -0.032 | 0.014 | 5.147 | 1 | 0.023 |
| PRS-BD | 0.042 | 0.118 | 0.128 | 1 | 0.720 |
| PRS-SZ | 0.147 | 0.115 | 1.611 | 1 | 0.204 |
| PRS-MDD | 0.010 | 0.120 | 0.008 | 1 | 0.931 |
| PRS-ADHD | -0.189 | 0.114 | 2.761 | 1 | 0.097 |
| Constant | -0.223 | 2.783 | 0.006 | 1 | 0.936 |

**S2e) Lifetime number of mood episodes (n=816)**

| Variables | Beta | Standard Error | Wald | df | p |
| --- | --- | --- | --- | --- | --- |
| Age | 0.017 | 0.0033 | 25.688 | 1 | 4.10^-7^ |
| Sex | 0.049 | 0.0784 | 0.396 | 1 | 0.529 |
| BD type | 0.058 | 0.0774 | 0.569 | 1 | 0.451 |
| MADRS | 0.008 | 0.0042 | 3.889 | 1 | 0.049 |
| PRS-BD | 0.057 | 0.0414 | 1.874 | 1 | 0.171 |
| PRS-SZ | -0.080 | 0.0413 | 3.778 | 1 | 0.052 |
| PRS-MDD | 0.050 | 0.0437 | 1.287 | 1 | 0.257 |
| PRS-ADHD | -0.030 | 0.0389 | 0.603 | 1 | 0.437 |
| Constant | 1.020 | 1.0696 | 0.909 | 1 | 0.340 |

**S2f) Rapid cycling (n=760)**

| Variables | Beta | Standard Error | Wald | df | p |
| --- | --- | --- | --- | --- | --- |
| Age | 0.010 | 0.008 | 1.469 | 1 | 0.225 |
| Sex | 0.003 | 0.208 | 0.000 | 1 | 0.988 |
| BD type | 0.017 | 0.207 | 0.007 | 1 | 0.934 |
| MADRS | 0.042 | 0.010 | 16.540 | 1 | **0.00005** |
| PRS-BD | -0.104 | 0.111 | 0.878 | 1 | 0.349 |
| PRS-SZ | 0.035 | 0.108 | 0.108 | 1 | 0.742 |
| PRS-MDD | 0.259 | 0.115 | 5.044 | 1 | 0.025 |
| PRS-ADHD | 0.016 | 0.110 | 0.021 | 1 | 0.885 |
| Constant | 0.191 | 2.501 | 0.006 | 1 | 0.939 |

**S2g) Suicidal attempt (n=828)**

| Variables | Beta | Standard Error | Wald | df | p |
| --- | --- | --- | --- | --- | --- |
| Age | 0.004 | 0.006 | 0.399 | 1 | 0.528 |
| Sex | 0.636 | 0.160 | 15.859 | 1 | **0.00007** |
| BD type | 0.185 | 0.153 | 1.464 | 1 | 0.226 |
| MADRS | 0.017 | 0.008 | 4.207 | 1 | 0.040 |
| PRS-BD | 0.046 | 0.081 | 0.327 | 1 | 0.567 |
| PRS-SZ | 0.011 | 0.080 | 0.020 | 1 | 0.887 |
| PRS-MDD | 0.271 | 0.086 | 9.983 | 1 | 0.002 |
| PRS-ADHD | -0.060 | 0.080 | 0.558 | 1 | 0.455 |
| Constant | -0.953 | 2.021 | 0.222 | 1 | 0.637 |

**S2h) Current smoking (n=811)**

| Variables | Beta | Standard Error | Wald | df | p |
| --- | --- | --- | --- | --- | --- |
| Age | -0.030 | 0.006 | 23.355 | 1 | <0.001 |
| Sex | -0.166 | 0.153 | 1.180 | 1 | 0.277 |
| BD type | -0.169 | 0.150 | 1.274 | 1 | 0.259 |
| MADRS | 0.009 | 0.008 | 1.129 | 1 | 0.288 |
| PRS-BD | 0.065 | 0.081 | 0.637 | 1 | 0.425 |
| PRS-SZ | -0.130 | 0.079 | 2.698 | 1 | 0.100 |
| PRS-MDD | 0.064 | 0.084 | 0.571 | 1 | 0.450 |
| PRS-ADHD | 0.152 | 0.079 | 3.702 | 1 | 0.054 |
| Constant | 2.155 | 1.992 | 1.170 | 1 | 0.279 |

**S2i) Lifetime alcohol use disorders (n=753)**

| Variables | Beta | Standard Error | Wald | df | p |
| --- | --- | --- | --- | --- | --- |
| Age | -0.003 | 0.007 | 0.147 | 1 | 0.702 |
| Sex | -0.666 | 0.177 | 14.101 | 1 | **0.0002** |
| BD type | 0.076 | 0.179 | 0.183 | 1 | 0.669 |
| MADRS | 0.036 | 0.009 | 14.334 | 1 | **0.0001** |
| PRS-BD | -0.049 | 0.096 | 0.262 | 1 | 0.609 |
| PRS-SZ | -0.062 | 0.094 | 0.437 | 1 | 0.509 |
| PRS-MDD | 0.235 | 0.100 | 5.503 | 1 | 0.019 |
| PRS-ADHD | 0.129 | 0.094 | 1.900 | 1 | 0.168 |
| Constant | -0.360 | 2.267 | 0.025 | 1 | 0.874 |

**S2j) Lifetime cannabis use disorders (n=753)**

| Variables | Beta | Standard Error | Wald | df | p |
| --- | --- | --- | --- | --- | --- |
| Age | -0.046 | 0.009 | 27.216 | 1 | **2.10^-7^** |
| Sex | -0.856 | 0.188 | 20.739 | 1 | **5.10^-6^** |
| BD type | -0.362 | 0.191 | 3.597 | 1 | 0.058 |
| MADRS | 0.022 | 0.010 | 4.832 | 1 | 0.028 |
| PRS-BD | -0.054 | 0.103 | 0.279 | 1 | 0.598 |
| PRS-SZ | -0.127 | 0.101 | 1.566 | 1 | 0.211 |
| PRS-MDD | 0.112 | 0.107 | 1.110 | 1 | 0.292 |
| PRS-ADHD | 0.022 | 0.099 | 0.047 | 1 | 0.828 |
| Constant | 3.109 | 2.368 | 1.724 | 1 | 0.189 |

**S2k) Lifetime anxiety disorders (n=739)**

| Variables | Beta | Standard Error | Wald | df | p |
| --- | --- | --- | --- | --- | --- |
| Age | -0.011 | 0.006 | 3.116 | 1 | 0.078 |
| Sex | 0.391 | 0.163 | 5.724 | 1 | 0.017 |
| BD type | 0.301 | 0.158 | 3.605 | 1 | 0.058 |
| MADRS | 0.052 | 0.009 | 30.785 | 1 | **3.10^-8^** |
| PRS-BD | -0.094 | 0.087 | 1.179 | 1 | 0.277 |
| PRS-SZ | -0.056 | 0.084 | 0.439 | 1 | 0.507 |
| PRS-MDD | 0.147 | 0.090 | 2.630 | 1 | 0.105 |
| PRS-ADHD | 0.053 | 0.084 | 0.394 | 1 | 0.530 |
| Constant | -2.336 | 2.101 | 1.236 | 1 | 0.266 |

*BD: bipolar disorder. MADRS: Montgomery Asberg Depression Rating Scale. PRS-BD: polygenic risk score for bipolar disorder. PRS-SZ: polygenic risk score for schizophrenia. PRS-MDD: polygenic risk score for major depressive disorder. PRS-ADHD: polygenic risk score for attention deficit with hyperactivity disorder*

*All models were adjusted for the first six principal components of the population’s genetic substructure.*

***In bold: p values significant after correction for multiple testing (p<0.0006)***

**Supplementary table S3: non-parametric correlations between childhood and adulthood dimensions**

| Variables |  | WURS | AIM | ALS | BIS10 | BDHI Attitudinal | BDHI Motor |
| --- | --- | --- | --- | --- | --- | --- | --- |
| CTQ | Rho | 0.39 | 0.20 | 0.26 | 0.20 | 0.35 | 0.26 |
|  | p | **2.10^-30^** | **1.10^-8^** | **1.10^-13^** | **7.10^-9^** | **7.10^-23^** | **4.10^-13^** |
|  | n | 782 | 783 | 774 | 784 | 755 | 749 |
| WURS | Rho |  | 0.37 | 0.49 | 0.47 | 0.46 | 0.51 |
|  | p |  | **1.10^-26^** | **2.10^-49^** | **1.10^-44^** | **1.10^-40^** | **1.10^-50^** |
|  | n |  | 776 | 767 | 777 | 750 | 745 |
| AIM | Rho |  |  | 0.61 | 0.41 | 0.43 | 0.46 |
|  | p |  |  | **7.10^-80^** | **2.10^-33^** | **7.10^-36^** | **4.10^-41^** |
|  | n |  |  | 773 | 782 | 757 | 751 |
| ALS | Rho |  |  |  | 0.48 | 0.57 | 0.58 |
|  | p |  |  |  | **3.10^-45^** | **3.10^-65^** | **3.10^-68^** |
|  | n |  |  |  | 775 | 748 | 743 |
| BIS10 | Rho |  |  |  |  | 0.36 | 0.40 |
|  | p |  |  |  |  | **1.10^-24^** | **5.10^-30^** |
|  | n |  |  |  |  | 756 | 750 |
| BDHI Attitudinal | Rho |  |  |  |  |  | 0.54 |
|  | p |  |  |  |  |  | **4.10^-58^** |
|  | n |  |  |  |  |  | 755 |

*AIM: Affect Intensity Measure; ALS: Affective Lability Scale; BDHI Attitudinal: Buss Durkee Hostility Inventory Attitudinal component; BDHI Motor: Buss Durkee Hostility Inventory Motor component; BIS: Barrat Impulsivity Scale. CTQ: Childhood Trauma Questionnaire. WURS: Wender Utah rating Scale.*

***In bold: p values significant after correction for multiple testing (p<0.0006)***

**Tables S4a to S4f: Associations between PRS, childhood dimensions, and adulthood dimensions (multivariate analyses)**

**S4a) Childhood Trauma Questionnaire (log transformed) (n=799)**

| Variables | Unstandardized Coefficients | |  |  |  |
| --- | --- | --- | --- | --- | --- |
|  | Beta | Standard Error | t | p | VIF |
| Constant | 1.818 | 0.126 | 14.486 | <0.001 |  |
| Age | 0.001 | 0.000 | 3.023 | 0.003 | 1.027 |
| Sex | 0.031 | 0.009 | 3.303 | 0.001 | 1.038 |
| BD type | 0.006 | 0.009 | 0.619 | 0.536 | 1.075 |
| MADRS | 0.003 | 0.001 | 5.061 | **5.10^-7^** | 1.100 |
| PRS-BD | -0.001 | 0.005 | -0.246 | 0.806 | 1.233 |
| PRS-SZ | -0.004 | 0.005 | -0.768 | 0.443 | 1.197 |
| PRS-MDD | 0.010 | 0.005 | 2.011 | 0.045 | 1.322 |
| PRS-ADHD | 0.019 | 0.005 | 4.004 | **7.10^-5^** | 1.185 |

**S4b) Wender Utah Rating Scale (n=790)**

| Variables | Unstandardized Coefficients | |  |  |  |
| --- | --- | --- | --- | --- | --- |
|  | Beta | Standard Error | t | p | VIF |
| Constant | 67.989 | 18.051 | 3.767 | <0.001 |  |
| Age | -0.117 | 0.054 | -2.177 | 0.030 | 1.026 |
| Sex | -4.253 | 1.347 | -3.157 | 0.002 | 1.035 |
| BD type | 3.542 | 1.330 | 2.663 | 0.008 | 1.076 |
| MADRS | 0.366 | 0.076 | 4.811 | **2.10^-6^** | 1.097 |
| PRS-BD | -0.851 | 0.719 | -1.183 | 0.237 | 1.237 |
| PRS-SZ | -1.329 | 0.703 | -1.892 | 0.059 | 1.194 |
| PRS-MDD | 1.332 | 0.736 | 1.809 | 0.071 | 1.333 |
| PRS-ADHD | 3.866 | 0.691 | 5.594 | **3.10^-8^** | 1.185 |

**S4c) Affect Intensity Measure (n=789)**

| Variables | Unstandardized Coefficients | |  |  |  |
| --- | --- | --- | --- | --- | --- |
|  | Beta | Standard Error | t | p | VIF |
| Constant | 2.933 | 0.623 | 4.704 | <0.001 |  |
| Age | -0.003 | 0.002 | -1.475 | 0.141 | 1.025 |
| Sex | 0.293 | 0.047 | 6.292 | **5.10^-10^** | 1.033 |
| BD type | 0.133 | 0.046 | 2.894 | 0.004 | 1.073 |
| MADRS | 0.018 | 0.003 | 6.801 | **2.10^-11^** | 1.099 |
| PRS-BD | -0.014 | 0.025 | -0.548 | 0.584 | 1.241 |
| PRS-SZ | -0.029 | 0.024 | -1.203 | 0.229 | 1.197 |
| PRS-MDD | 0.073 | 0.025 | 2.873 | 0.004 | 1.315 |
| PRS-ADHD | 0.070 | 0.024 | 2.903 | 0.004 | 1.182 |

**S4d) Affective Lability Scale (n=779)**

| Variables | Unstandardized Coefficients | |  |  |  |
| --- | --- | --- | --- | --- | --- |
|  | Beta | Standard Error | t | p | VIF |
| Constant | 1.228 | .588 | 2.089 | 0.037 |  |
| Age | -0.005 | 0.002 | -2.824 | 0.005 | 1.026 |
| Sex | 0.079 | 0.044 | 1.792 | 0.074 | 1.034 |
| BD type | 0.227 | 0.043 | 5.238 | **2.10^-7^** | 1.074 |
| MADRS | 0.024 | 0.003 | 9.490 | **3.10^-20^** | 1.097 |
| PRS-BD | -0.020 | 0.023 | -0.865 | 0.387 | 1.220 |
| PRS-SZ | -0.040 | 0.023 | -1.770 | 0.077 | 1.193 |
| PRS-MDD | 0.073 | 0.024 | 3.074 | 0.002 | 1.311 |
| PRS-ADHD | 0.081 | 0.023 | 3.543 | **4.10^-4^** | 1.186 |

**S4e) Barratt Impulsivity Scale (n=788)**

| Variables | Unstandardized Coefficients | |  |  |  |
| --- | --- | --- | --- | --- | --- |
|  | Beta | Standard Error | t | p | VIF |
| Constant | 90.335 | 10.970 | 8.235 | <0.001 |  |
| Age | -0.109 | 0.032 | -3.360 | 0.0008 | 1.028 |
| Sex | -0.371 | 0.817 | -0.454 | 0.650 | 1.037 |
| BD type | 1.023 | 0.802 | 1.275 | 0.203 | 1.077 |
| MADRS | 0.303 | 0.046 | 6.545 | **1.10^-10^** | 1.100 |
| PRS-BD | -0.454 | 0.432 | -1.051 | 0.293 | 1.235 |
| PRS-SZ | -1.150 | 0.421 | -2.733 | 0.006 | 1.196 |
| PRS-MDD | 0.602 | 0.441 | 1.367 | 0.172 | 1.327 |
| PRS-ADHD | 1.966 | 0.418 | 4.701 | **3.10^-6^** | 1.193 |

**S4f) Buss and Durkee Hostility Inventory (Attitudinal component) (n=761)**

| Variables | Unstandardized Coefficients | |  |  |  |
| --- | --- | --- | --- | --- | --- |
|  | Beta | Standard Error | t | p | VIF |
| Constant | 7.021 | 3.951 | 1.777 | 0.076 |  |
| Age | -0.050 | 0.012 | -4.258 | **0.00002** | 1.026 |
| Sex | 0.431 | 0.296 | 1.454 | 0.146 | 1.035 |
| BD type | 0.391 | 0.290 | 1.348 | 0.178 | 1.069 |
| MADRS | 0.168 | 0.017 | 9.899 | **8.10^-22^** | 1.088 |
| PRS-BD | -0.296 | 0.158 | -1.882 | 0.060 | 1.235 |
| PRS-SZ | -0.277 | 0.153 | -1.809 | 0.071 | 1.186 |
| PRS-MDD | 0.479 | 0.162 | 2.967 | 0.003 | 1.347 |
| PRS-ADHD | 0.543 | 0.153 | 3.555 | **4.10^-4^** | 1.201 |

**S4g) Buss and Durkee Hostility Inventory (Motor component) (n=755)**

| Variables | Unstandardized Coefficients | |  |  |  |
| --- | --- | --- | --- | --- | --- |
|  | Beta | Standard Error | t | p | VIF |
| Constant | 18.672 | 7.536 | 2.478 | 0.013 |  |
| Age | -0.108 | 0.023 | -4.814 | **2.10^-6^** | 1.029 |
| Sex | 0.169 | 0.566 | 0.299 | 0.765 | 1.037 |
| BD type | 1.941 | 0.555 | 3.498 | **0.0005** | 1.068 |
| MADRS | 0.166 | 0.032 | 5.133 | **4.10^-7^** | 1.089 |
| PRS-BD | -0.359 | 0.300 | -1.194 | 0.233 | 1.237 |
| PRS-SZ | -0.147 | 0.294 | -0.502 | 0.616 | 1.185 |
| PRS-MDD | 0.783 | 0.309 | 2.532 | 0.012 | 1.343 |
| PRS-ADHD | 1.277 | 0.292 | 4.379 | **1.10^-5^** | 1.195 |

*BD: bipolar disorder. MADRS: Montgomery Asberg Depression Rating Scale. PRS-BD: polygenic risk score for bipolar disorder. PRS-SZ: polygenic risk score for schizophrenia. PRS-MDD: polygenic risk score for major depressive disorder. PRS-ADHD: polygenic risk score for attention deficit with hyperactivity disorder*

*VIF: variance inflation factor*

*All models were adjusted for the first six principal components of the population’s genetic substructure.*

***In bold: p values significant after correction for multiple testing (p<0.0006)***

**Supplementary table S5a and S5b: Associations between childhood trauma questionnaire total score, clinical variables and comorbidities in BD**

***S5a) Associations between CTQ score, age at onset and lifetime number of mood episodes* (Spearman correlation tests)**

| Variables | CTQ total score | | |
| --- | --- | --- | --- |
|  | N | Rho | p |
| Age at onset | 765 | -0.10 | 0.006 |
| Lifetime mood episodes | 768 | 0.04 | 0.24 |

**S5b) Associations CTQ score, clinical variables and comorbidities (Mann-Whitney tests)**

| Variables | N | Median | IQR | Median | IQR | p |
| --- | --- | --- | --- | --- | --- | --- |
|  |  | Absent | | Present | |  |
| Polarity at onset (depressive) | 825 | 37 | 31-47 | 37 | 31-47 | 0.53 |
| Psychotic symptoms at onset | 825 | 38 | 31-52 | 35 | 30-46 | 0.03 |
| Rapid cycling | 762 | 36 | 31-46 | 42 | 33-52 | 0.002 |
| Suicide attempt | 830 | 36 | 30-45 | 41 | 33-53 | **8.10^-9^** |
| Alcohol use disorders | 755 | 36 | 30-46 | 39 | 33-51 | 0.002 |
| Cannabis use disorders | 755 | 37 | 31-46 | 37 | 31-49 | 0.94 |
| Anxiety disorders | 741 | 35 | 30-43 | 40 | 32-51 | **5.10^-6^** |

*CTQ: Childhood Trauma Questionnaire.*

***In bold: p values significant after correction for multiple testing (p<0.0006)***

**Table S6: Test for the moderating effect of each PRS on the association between childhood trauma, the clinical presentation of BD and dimensions.**

| Variables | N | CTQ*PRS-BD | CTQ*PRS-SZ | CTQ*PRS-MDD | CTQ*PRS-ADHD |
| --- | --- | --- | --- | --- | --- |
| Clinical variables | | | | | |
| Suicide attempt | 779 | p=0.975 | p=0.279 | p=0.702 | p=0.368 |
| Anxiety disorders | 700 | p=0.747 | p=0.306 | p=0.612 | p=0.263 |
| Dimensions | | | | | |
| ADHD symptoms | 781 | p=0.369 | p=0.831 | p=0.790 | p=0.618 |
| Affective lability | 773 | p=0.624 | p=0.321 | p=0.537 | p=0.578 |
| Affect intensity | 782 | p=0.708 | p=0.225 | p=0.992 | p=0.778 |
| Impulsivity | 783 | p=0.158 | p=0.805 | p=0.483 | p=0.285 |
| Attitudinal hostility | 754 | p=0.688 | p=0.195 | p=0.860 | p=0.142 |
| Motor hostility | 748 | p=0.917 | p=0.257 | p=0.531 | p=0.451 |

*All models were adjusted for age, sex, BD type and MADRS.*

*For a purpose of clarity. only p values for interaction between each PRS and CTQ are displayed in the table (one model per PRS and per variable).*

*CTQ: Childhood Trauma Questionnaire (Log : log10 transformed). PRS: polygenic risk score. BD: bipolar disorder; SZ: schizophrenia; MDD: major depressive disorder; ADHD: attention deficit with hyperactivity disorder. MADRS: Montgomery Asberg Depression Rating Scale.*

**Table S7: Summary of specific indirect effects from PRS-ADHD and PRS-MDD to dimensions through CTQ and WURS**

| PRS | Mediator | Dimensions | Estimate | SE | Estimate/SE | p |
| --- | --- | --- | --- | --- | --- | --- |
| PRS-MDD | CTQ | BDHI Att | 0.024 | 0.009 | 2.690 | 0.007 |
| PRS-MDD | WURS | AIM | 0.048 | 0.019 | 2.470 | 0.014 |
| PRS-MDD | WURS | ALS | 0.052 | 0.020 | 2.565 | 0.010 |
| PRS-MDD | WURS | BDHI Att | 0.039 | 0.016 | 2.417 | 0.016 |
| PRS-MDD | WURS | BDHI Mot | 0.062 | 0.024 | 2.569 | 0.010 |
| PRS-MDD | WURS | BIS | 0.061 | 0.024 | 2.584 | 0.010 |
|  |  |  |  |  |  |  |
| PRS-ADHD | CTQ | BDHI Att | 0.020 | 0.008 | 2.620 | 0.009 |
| PRS-ADHD | WURS | AIM | 0.061 | 0.019 | 3.230 | 0.001 |
| PRS-ADHD | WURS | ALS | 0.067 | 0.021 | 3.207 | 0.001 |
| PRS-ADHD | WURS | BDHI Att | 0.049 | 0.016 | 3.160 | 0.002 |
| PRS-ADHD | WURS | BDHI Mot | 0.079 | 0.024 | 3.312 | 0.001 |
| PRS-ADHD | WURS | BIS | 0.078 | 0.024 | 3.313 | 0.001 |

*PRS: polygenic risk score. MDD: Major Depressive Disorder. ADHD: attention deficit with hyperactivity disorder. CTQ: Childhood Trauma Questionnaire. WURS: Wender Utah Rating Scale (childhood ADHD symptoms). AIM: Affect Intensity Measure; ALS: Affective Lability Scale; BDHI Att: Buss Durkee Hostility Inventory Attitudinal component; BDHI Mot: Buss Durkee Hostility Inventory Motor component; BIS: Barrat Impulsivity Scale. SE: standard error.*
